# Supplementary figures and images for: Phylogeny of PmCCD Gene Family and Expression Analysis of Flower Coloration and Stress Response in Prunus mume
Source: Int J Mol Sci. 2023 Sep 11;24(18):13950. doi: 10.3390/ijms241813950 (PMC10531161; doi:10.3390/ijms241813950)

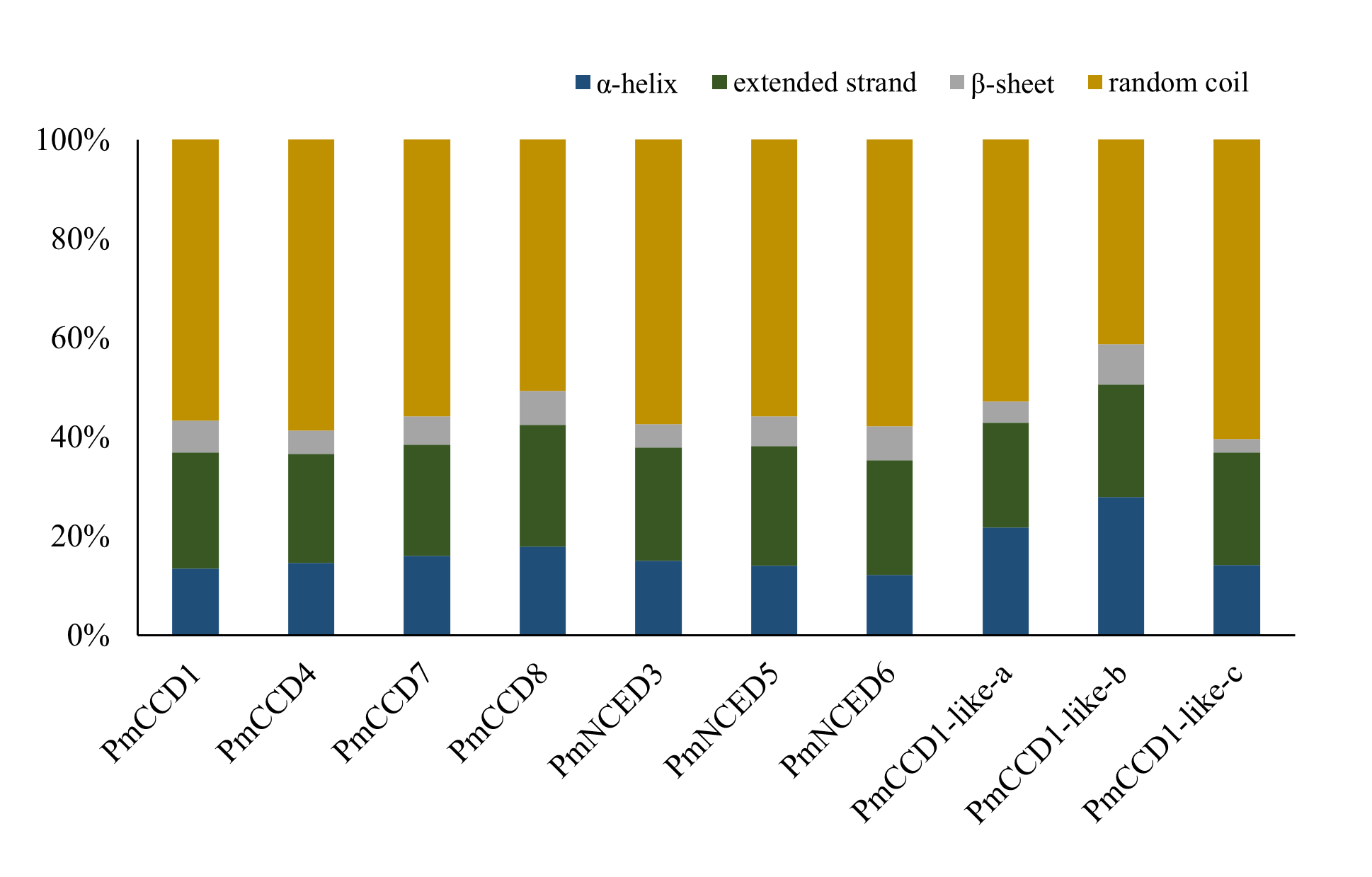

Supplement: Supplementary file 1 [file ijms-24-13950-s001.zip › Supplementary Figure 1.tif]

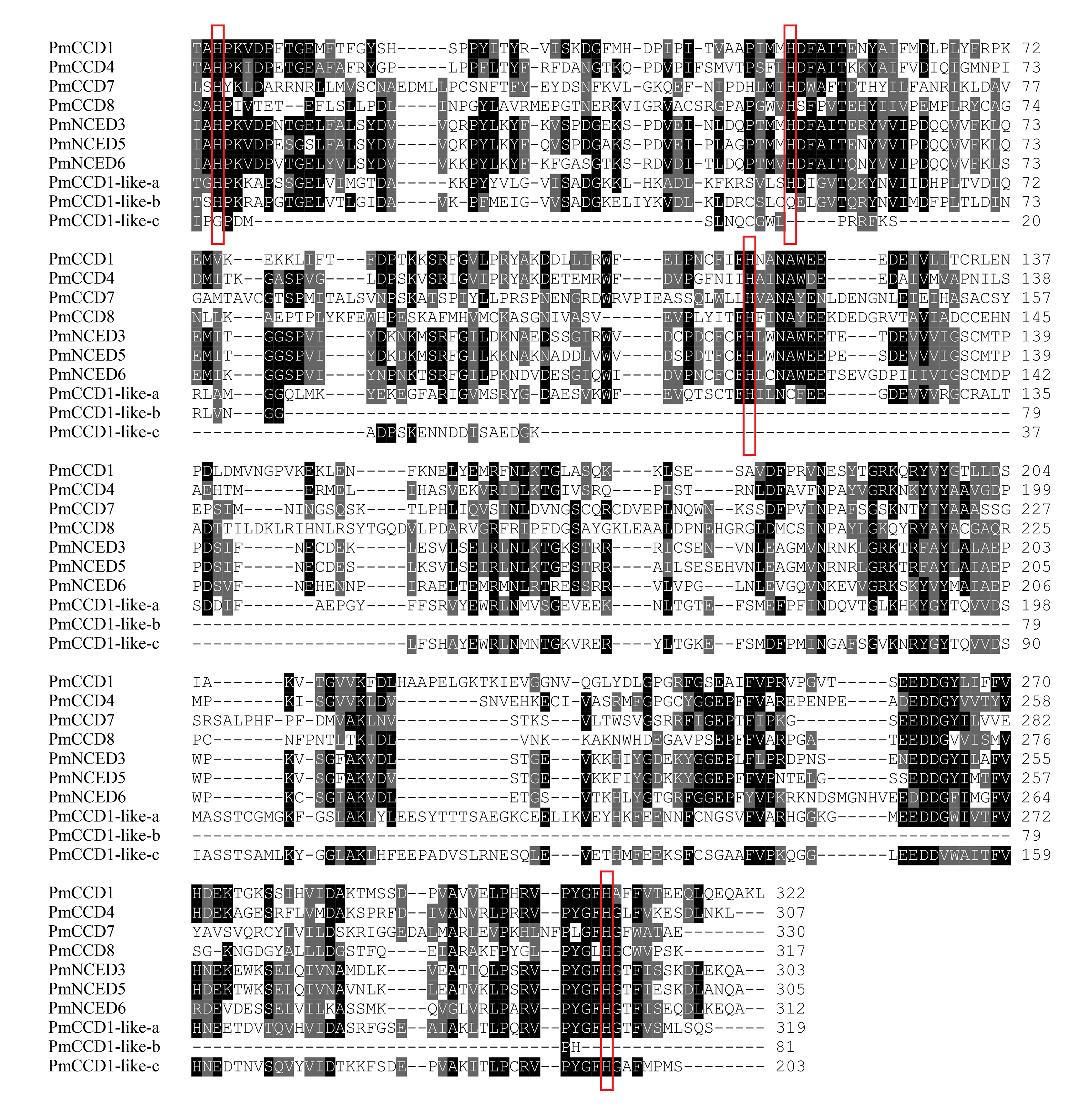

Supplement: Supplementary file 1 [file ijms-24-13950-s001.zip › Supplementary Figure 2.tif]
